# Tailored implementation for chronic diseases (TICD): A project protocol

**DOI:** 10.1186/1748-5908-6-103

**Published:** 2011-09-07

**Authors:** Michel Wensing, Andy Oxman, Richard Baker, Maciek Godycki-Cwirko, Signe Flottorp, Joachim Szecsenyi, Jeremy Grimshaw, Martin Eccles

**Affiliations:** 1 Scientific Institute for Quality of Healthcare, Nijmegen, Radboud University Nijmegen Medical Centre, Geert Grooteplein, Nijmegen, the Netherlands; 2 Global Health Unit, Norwegian Knowledge Centre for the Health Services, Olavsplass, Oslo, Norway; 3 Department of Health Sciences, University of Leicester, Princess Road West, Leicester, UK; 4 Department of Family and Community Medicine, Medical University of Lodz,, Kopcinskiego Lodz, Poland; 5 Department of General Practice and Health Services Research, University of Heidelberg Hospital, Vossstrasse, Heidelberg, Germany; 6 Clinical Epidemiology Program, Ottawa Health Research Institute, Carling Avenue, Administrative Building, Ottawa, Canada; 7 Institute of Health and Society, Newcastle University, Baddiley-Clark Building, Richardson Road, Newcastle upon Tyne, UK

## Abstract

**Background:**

The assumption underlying tailoring is that implementation interventions are most helpful if these effectively address the most important determinants of practice for improvement in the targeted setting. The aim of the Tailored Implementation For Chronic Diseases (TICD) project is to develop valid and efficient methods of tailoring implementation interventions to determinants of practice for knowledge implementation in chronic illness care.

**Methods:**

The TICD project has organized the planned empirical research in three work packages that follow the three main steps of tailoring: identification of determinants of healthcare practice, matching implementation interventions to identified determinants of practice, and applying and assessing the tailored implementation interventions. These three key steps of tailored implementation will be applied to targeted chronic conditions in five different healthcare systems: cardiovascular disease in the Netherlands, obesity in England, depression in Norway, chronic obstructive pulmonary disease in Poland, and multimorbidity in Germany. The design and interpretation of empirical research will be informed by systematic reviews of previous research on tailoring implementation interventions.

**Discussion:**

The TICD project will provide much needed evidence on the advantages and disadvantages of different methods of identifying important determinants of practice and selecting implementation strategies that take account of those. It will also provide five rigorous evaluations of tailored implementation interventions for five different chronic conditions.

## Background

Tailored implementation interventions are strategies that are designed to achieve desired changes in healthcare practice based on an assessment of determinants of healthcare practice [1]. Systematic tailoring entails three key steps: identification of the determinants of healthcare practice, designing implementation interventions appropriate to the determinants, and application and assessment of implementation interventions that are tailored to the identified determinants. While the process of 'tailoring' may be used refer to the second step only, in this paper it is used in a more comprehensive way to include these three steps. 'Tailored implementation interventions' is the short phrase for implementation interventions resulting from a tailoring process. Little research evidence is available regarding how tailoring is best done in relation to implementation interventions.

Determinants of healthcare practice are factors that might prevent or enable improvements. Such factors are sometimes referred to as barriers and enablers, barriers and facilitators, problems and incentives, or as moderators and mediators. These include factors that can be modified (*e.g*., knowledge of health professionals) and non-modifiable factors that can be used to target interventions (*e.g*., wider organizational structures). Determinants of current practice are included if they are relevant to achieving change. The factors can be related to professional behaviour, organisation of healthcare, and health system arrangements. They can also be related to patient behaviours that might prevent or enable healthcare improvements and characteristics of the social and political environment, which might constrain or enable efforts to improve health services. Factors may be pragmatically defined or linked to theoretical perspectives. Healthcare improvements include improvements in any healthcare setting (including primary and secondary care) and improvements in public health services as well as clinical services.

The assumption underlying tailoring is that implementation interventions are most helpful if these effectively address the most important determinants of practice for improvement in the targeted setting. This is consistent with a rational approach to clinical practice, where a diagnosis is made in order to guide the choice of treatment. The idea is also shared with a large number of theories and models for inducing behavioural and organizational change, which have been developed in various scientific disciplines such as motivational psychology, organisational science, and educational research [2]. Many descriptive studies of determinants of practice have been published in medical journals and an increasing number of implementation interventions have been labeled 'tailored implementation interventions.' Although tailoring implementation interventions to determinants of practice seems logical and has received growing attention, research evidence that tailored strategies are substantially more effective than other approaches is lacking [3].

Furthermore, it is unclear how best to identify important determinants of practice and how to match implementation interventions to those. A range of approaches is available for the different steps in tailoring, as will be outlined in the following section. It is unclear which ones are most appropriate. For example, a meta-regression analysis on 26 studies of tailored interventions did not identify impact of level of tailoring, rigour of barrier analysis, complexity of interventions, concealment of allocation, explicit utilisation of a theory when developing the intervention, and the reported presence or absence of administrative constraints [3]. The Tailored Implementation For Chronic Diseases (TICD) project aims to address this lack of research evidence by directly comparing alternative approaches in the tailoring process and by assessing the effectiveness of resulting tailored implementation interventions.

## Challenges regarding tailored implementation

The available research on tailored implementation interventions signals a number of challenges. Tailoring methods have been poorly described in published research. For example, a qualitative in-depth analysis of a heterogeneous set of 20 tailored interventions found that a wide variety of tailoring methods was used, methods were poorly described, and there was little matching between identified barriers for change and implementation interventions chosen [4]. Poor documentation reduces the possibility of learning from previous studies, makes it difficult to standardize methods, and inhibits the development of a shared knowledge base.

Although many studies of determinants of practice have now been published in healthcare journals, the validation of the measurement approaches is often limited. Whether theory-based or pragmatic, non-validated measures often are used in small studies. For example, many studies were based on physician-reported barriers for change using poorly developed questionnaires. Furthermore, much research is cross-sectional and precedes the implementation process. It is possible that identified determinants of practice are not relevant to the actual implementation process, and during the implementation process determinants of practice may be present that were not identified.

The different methods and models for tailoring reflect opposing approaches to implementation science, which have been advocated by implementation scientists in the TICD consortium. Eccles *et al*. have argued for wider use of theory in implementation research, both for intervention development and for evaluations of intervention effectiveness [5]. A wide range of theories is available, and personal preference rather than research evidence seems to guide the choice of theory [2]. Theory-based approaches are explicitly linked to one or more specific theories, such as the Theory of Planned Behavior or the Diffusion of Innovation theory, and derive relevant factors and methods from such theories. Oxman *et al*., on the other hand, have argued for a pragmatic and empirical approach to implementation science [6]. Pragmatic models specify a list of potentially relevant factors, but do not embed these in a comprehensive theoretical framework. Furthermore, different theory-based approaches compete with each other, just as different pragmatic approaches compete with each other. Evidence to support any approach is limited.

While there seems to be broad consensus on the value of tailoring implementation interventions to local determinants of practice, another issue is what 'local' means. Its meaning varies from 'a specific project' or 'a specific health profession' to 'a specific care provider' or 'a specific aspect of the behaviour of that care provider' (*e.g*., weight monitoring in diabetic patients). The appropriate aggregation level depends on the generalizability of the identified determinants of practice. The choice of aggregation level also may have implications for costs, because tailoring an implementation intervention to each individual health professional is likely to be more resource consuming than tailoring to higher aggregation levels. Tailoring to individual health professionals would be less costly if more intensive and expensive interventions were only used for individuals where they were thought to be needed, rather than for everyone.

Another area of uncertainty concerns the timing of the tailoring process. Tailoring is mostly thought of as an analysis of determinants of practice, and matching of implementation interventions to those determinants of practice, before implementation interventions are actually applied. But this may be too early in some situations, such as in the case of a very innovative technology, because the target group may have to experience the innovation first. In other situations, it may be effective to repeat the tailoring process after an implementation intervention has been started, in order to guide modifications of the implementation process.

## Aim and objectives

The aim of the TICD project is to develop valid and efficient methods of tailoring implementation interventions to determinants of practice for knowledge implementation in chronic illness care. Four key objectives have been defined:

1. To review research evidence regarding approaches to tailoring knowledge implementation in healthcare practice.

2. To test different approaches for identifying determinants of healthcare practice in chronic illness care.

3. To test different approaches for matching implementation interventions to identified determinants of healthcare practice in chronic illness care.

4. To assess the effectiveness of tailored implementation interventions in chronic illness care and the role of hypothesized determinants of healthcare practice.

## Methods

The TICD project has organized the planned empirical research in three work packages that follow the three main steps of tailoring: identification of determinants of healthcare practice, matching implementation interventions to identified determinants of practice, and applying and assessing the tailored implementation interventions. These three key steps of tailored implementation will be applied to targeted chronic conditions in five different healthcare systems: cardiovascular disease in the Netherlands, obesity in England, depression in Norway, chronic obstructive pulmonary disease in Poland, and multimorbidity in Germany. The design and interpretation of empirical research will be informed by systematic reviews of previous research on tailoring implementation interventions. Ethical approval will be sought for each of the studies separately according to national regulations.

### Systematic reviews

We will prepare systematic reviews of both descriptive and evaluative studies of different approaches to identifying determinants of practice, and approaches to matching implementation interventions to determinants of practice. These reviews will be undertaken the first year of the project and updated during the project.

### Approaches to identifying determinants of practice and for matching interventions

We will include both descriptive and evaluative studies of methods that have been used to identify barriers or enablers to changing health professional practice or matching interventions to determinants of practice. We will use text words and index terms from published papers that we already have on file to construct search strategies for Medline and Embase. We will conduct citation searches (ISI and Google) and search for related articles in PubMed using key background papers and relevant included studies. We will screen the reference lists of key background documents and relevant studies, and we will contact key informants, including the authors of key background documents and included studies.

Two reviewers will independently read the titles and abstracts resulting from the search process and eliminate any obviously irrelevant studies. We will retrieve the full text of potentially relevant studies. Two reviewers will then assess each retrieved study using the selection criteria. Studies meeting all of the selection criteria will be included. Disagreements will be resolved by consensus of all of the reviewers. Data will be extracted independently from each included study by two of the review authors using a standard data extraction form. Discrepancies will be resolved by checking against the study report and, if needed, discussed with the other review authors. We will contact the investigators to collect information that is missing from study reports. For each method, we will extract a detailed description of the method, time, and resources required to apply the method, the advantages and disadvantages of the method, and the evidence or logical arguments supporting those. The Cochrane Effective Practice and Organisation of Care (EPOC) risk of bias approach will be used for evaluative studies. We will assess the risk of bias in descriptive studies using the following criteria:

1. The methods for data collection were appropriate for the purpose of the study.

2. The sources of information were appropriate for the purpose of the study.

3. The methods used to analyze the data were appropriate for the purpose of the study.

4. The linkages are transparent between the data that were reported and inferences.

We will construct tables summarizing key advantages and disadvantages of each method to facilitate comparisons across different methods. We will summarize the strengths and weaknesses of each method, ways in which different methods complement each other and could be potentially combined, and gaps in evidence to support potential strengths and weaknesses of each method.

### Checklist for determinants of practice

Terms such as checklist, framework, and taxonomy have different meanings. We use the term 'checklist' here as a generic term for any system for identifying and classifying determinants of change in practice.

We will identify checklists in papers included in the review described above. We will contact key informants, including members of an advisory group and the authors of key background documents and included articles, to identify additional checklists. We will review the advantages and disadvantages of existing checklists and compile a comprehensive list of factors included in those checklists and the ways in which factors are grouped ('dimensions'). We will add factors and dimensions that are not included in existing checklists based on input from an international advisory group. We will then organize and group factors into a draft checklist.

We will compile a list of attributes that a checklist of determinants of practice should have by circulating a draft list to the advisory group and revising it based on their input. We will ask the advisory group to appraise the draft checklist using the revised list of desirable attributes as criteria. We will revise the checklist based on their feedback and send the revised checklist with the compiled feedback to the advisory group, requesting them to appraise the revised checklist using the same criteria. The resulting checklist will be tested in work packages two through four, which are described below.

### New updates of Cochrane review on tailored interventions

We will update the Cochrane review of tailored interventions [3] that assessed the effectiveness of tailored implementation strategies in improving professional practice and healthcare outcomes. The review includes a comparison of interventions tailored to address identified barriers to change with no intervention or an intervention(s) not tailored to the barriers. We will undertake this analysis for two subsets of the studies, one in which the control group received no intervention and the other in which the control was a non-tailored intervention. We will also undertake an investigation of heterogeneity of the effectiveness of tailored interventions to identify factors important to consider when designing and implementing a tailored intervention. We will also compare interventions targeted at both individual and social or organisational barriers compared with interventions that are targeted at only individual barriers. This review was last updated in 2009 [3].

### Methods for identification of determinants of healthcare practice

In this work package, we will evaluate different methods for identifying determinants of practice. We will first describe gaps or deficiencies in healthcare for the studied chronic conditions by drawing on publications and available datasets. Having defined the strengths and weaknesses of care, we will select up to four methods for identifying determinants of practice that appear to explain the deficiencies in care. Finally, we will compare methods and their findings to determine which methods are most appropriate to use, and to which contexts and settings they are most applicable.

### Inventory of current practices

In this stage, we define the healthcare problem to be investigated. This inventory will identify the performance gaps and define goals for improvement, and thus set the stage for the following steps. A comprehensive and up-to-date inventory of current practice in the care of the targeted chronic condition will be made by each of the participants. Although they will focus on research in their own country in order to provide evidence about the gaps and deficiencies in care in that country, the inventory will take account of evidence from other countries in order to set the findings in an international context. In each participant country, the inventory will address the specific, targeted condition for that country. For each condition, we will identify the national guidelines or key recommendations applicable in each of the participant countries. The following approaches will be used:

1) Review of published observational research on adherence to guidelines or aspects of care of the targeted chronic condition, using searches in electronic bibliographic databases, national journals, and national conferences; we will apply a standardized search strategy that will ensure consistency across targeted chronic conditions, and between participant countries. Whilst the review will not be of trials of clinical interventions, we will employ systematic approaches for the searches and assessment of articles for inclusion. Data from the included articles will be extracted to a table, and the findings summarized in a narrative review that will compare evidence on what care is delivered with the prevailing guideline recommendations.

2) Analysis of available epidemiological or public health datasets, or data from surveillance networks that continuously collect data on disease incidence and prevalence as well as healthcare provided. For example, some practice level data on management of obesity are available in England; in different countries and for the different clinical conditions, a variety of data are available. The analyses will be descriptive, and will be used to highlight discrepancies between recommended care and actual care.

3) In order to check our interpretation of our evidence and data reviews, we will interview up to five key informants on each country. The informants will be selected for their knowledge of policy and practice of the targeted condition in the context of their country setting. The interview will enquire whether our interpretation of the gaps and deficiencies in performance reflect their own knowledge and experience. If differences between our interpretation and the views of the key informants are identified, we will re-visit the evidence and data to understand the inconsistencies.

### Selection of methods

We will use a structured approach to select up to four methods for identifying determinants of practice. The TICD participants and members of the scientific advisory board will be involved in this process that will employ a two- or three-stage modified Delphi procedure to identify consensus on the most appropriate methods to select. In the Delphi procedure, respondents will be asked to rate the suitability of the candidate methods using a set of criteria that will include the extent to which the methods identify a comprehensive range of determinants, whether they identify the most relevant determinants, whether there is evidence of their validity, and whether they feasible to employ and of reasonable cost. The Delphi procedure will also seek consensus on the extent to which a checklist should be used to assist in the identification of determinants, and the extent to which behavioural theory should inform the use of the methods.

### Comparative evaluation

Head-to-head comparisons of barrier identification methods will be conducted in each country for the targeted chronic conditions (*i.e*., five discrete but related studies, each addressing one of cardiovascular risk, obesity, mental health, multi-morbidity, or asthma). An internationally standardized protocol for these studies will be developed to ensure that the chosen methods are used consistently across countries. In each country, up to four methods will be used. Because there is no reference standard already known as a valid method to identify determinants of practice, we will take as the reference, for each condition, the total of all determinants identified by all methods combined. Brainstorming will be used as the reference methods involving (lowest) cost and time investment.

The setting of these studies will be dictated by the particular condition, and generally will involve primary and secondary care services. The study subjects will be clinicians, patients, managers or content experts. The number of participants in each study group is related to the method used; *e.g*., a survey in health professionals requires a larger sample than a brainstorming session with five to ten clinicians. Because the selected methods may be more appropriate for certain settings (for example, the team or organization level), random allocation of methods across settings would be not be helpful. Selection of methods will be made on explicit predictions of which method is likely to suit which condition and setting. For example, if care is team based, observation of teamwork, process mapping, or focus group methods may be more appropriate than individual interviews.

In each chronic condition, we aim to spread the selected methods over aggregation levels (health professional, team, organization) and orientation (explorative/pragmatic versus theory-orientated). One of the methods will be consistent across conditions, and we plan to use brainstorming in this role. A standard brainstorming session with pragmatic analysis of data represents a low cost, low intensity method. All other methods involve a greater degree of primary data collection and analysis, and therefore although brainstorming is not a no-intervention control, it offers a minimal method against which more intensive methods may be compared. Measures of process and outcomes will be standardized. The analysis will compare methods in terms of process (the time, resources, and expertise required), and outcomes (the range and completeness of determinants of practice identified, consistency of factors across methods, and whether the method highlighted the most salient determinants of practice as identified by the combined methods).

### Process evaluations

We will undertake a process evaluation in each country to describe the feasibility of use of the methods and check the fidelity of use of the methods. The research teams in each country will maintain a diary to record the required time and document any difficulties in applying the methods, any deviations from the recommended procedures for each method, and any other problems or positive experiences that occurred. The project diaries will be supplemented by interviews of each country researcher led by the lead of this work package in order to explore any emerging issues in depth. The findings of the diaries and interviews will be combined to create a report on the use of each method.

### Matching implementation interventions to identified determinants of practice

This work package focuses on the logical next step in tailoring, which is linking implementation interventions to identified determinants of practice. First, the list of determinants of practice will be standardized to set the stage for the next steps. Then, up to four methods will be selected for linking interventions to these determinants of practice. Some of these methods may be naturally linked to approaches for identifying determinants of practice, *e.g*., identified in the same questionnaire or focus group interview. While the strongest evidence for the usefulness of matching interventions will be provided by the planned evaluations of resulting tailored implementation interventions, this work package will examine outcomes specifically related to the matching methods.

### Standardization of determinants of practice

The identified determinants of practice for knowledge implementation in the targeted chronic diseases, resulting from the different approaches in the previous work package, will be standardized. This is done to provide an equal starting point for the research planned in this work package. In this process, we will take the validity and generalizability of the findings into account.

### Selection of methods

We will select up to four methods for matching implementation interventions to identified determinants of practice identified in the previous work package. The methods will be selected for testing, using a structured process. The process will employ a two- or three-stage modified Delphi procedure to identify consensus on the most appropriate methods to test. In the Delphi procedure, respondents will be asked to rate the suitability of the candidate methods using a set of criteria that will include the extent to which the methods can be linked to specific factors, whether there is evidence of their effectiveness, and whether they are efficient and feasible to employ. Depending on the number and nature of the methods identified in the literature reviews, in the Delphi ratings process we will group the methods according to whether they are pragmatic or theory based, and whether they address individual, team, or organization levels. Consequently, it will be possible to select the most highly rated methods from these categories.

### Comparative evaluations

Head-to-head comparisons of barrier identification methods will be conducted in each country for the targeted chronic condition (*i.e*., discrete but related studies). An internationally standardized protocol for these studies will guide these studies. The comparisons will be designed as comparative evaluations. One of the methods will be consistent across conditions, and we plan to use a short brainstorming session in this role. We will take care that study groups cannot influence each other during the study (to avoid contamination), that 'interventions' to match implementation interventions are well defined and implemented, and that measures for evaluation are standardized.

The setting of these evaluation trials will be dictated by the particular condition, and generally will involve primary and secondary care services. The study subjects will be clinicians, patients, managers, or content experts. Because the selected methods may be more appropriate for specific settings (for example, the team or organization level), random allocation of methods across settings would not be helpful. Selection of methods will be made on explicit predictions of which method is likely to suit which condition and setting. In each chronic condition, we plan to test up to four different methods, spread over aggregation levels (health professional, team, organization) and orientation (explorative/pragmatic versus theory-orientated). A pragmatic brainstorm session involving experienced clinicians represents a low-cost, low-intensity method. All other methods involve a greater degree of primary data collection and analysis, and therefore although brainstorming is not a no-intervention control, it offers a minimal method against which more intensive methods will be compared.

Measures include a log of activities and time investment, and documentation on the prioritized implementation interventions. The analysis will compare methods in terms of process (the time, resources, and expertise required), and outcomes (the range and completeness of interventions identified, consistency of interventions, and whether the method highlighted the most salient interventions as compared to the combined results of the different methods). The impact of chosen interventions will be studied in the trials, which are described below.

### Process evaluations

We will undertake a process evaluation in each country to describe the feasibility of use of the methods and check the fidelity of use of the methods. The research teams in each country will maintain a diary to record the required time and document any difficulties in applying the methods, any deviations from the recommended procedures for each method, and any other problems or positive experiences that occurred. The project diaries will be supplemented by interviews of each country research led by the lead of this work package in order to explore any emerging issues in depth. The findings of the diaries and interviews will be combined to create a report on the use of each method.

### Effectiveness of tailored interventions

In this final piece of empirical research, we will assess the effectiveness of the resulting tailored implementation interventions that were derived from the previous work package. Depending on feasibility, the studies will involve subjects who were also involved in previous work packages or newly recruited subjects. A detailed study protocol will be elaborated according to relevant guidelines, *e.g*., CONSORT, STROBE. Trials will be registered in an internationally recognized register. In this section an outline of the planned research is provided. First, we will make a final choice of implementation interventions and develop research protocols for each of the different (clusters) of chronic conditions. The research protocols will be internationally standardized with respect to methods and measures where possible to enhance comparability of study findings. We aim for cluster-randomized trials, when feasible. The strongest possible alternative design will be used, when cluster-randomized trials are not feasible (*e.g*., interrupted time series or controlled before-after comparisons). Measures related to identified determinants of practice will be included to explore their potential role in improving healthcare practice. Finally, we will apply observational and qualitative methods (surveys, interviews, focus groups) including patients and professionals as process evaluation.

### Selection of implementation interventions

Based on the results of the work package focused on matching interventions to determinants of practice, a list of implementation interventions will be made for each of the five targeted chronic conditions. Tailoring interventions implies that we cannot specify the interventions *a priori *in a standardized way. We expect to assess complex interventions consisting of several active components on different levels *e.g*., on the individual and organizational levels. Participants (practitioners and patients) may be different from those involved in the previous work packages, which may result in a different choice of implementation interventions compared to the previous work package. Furthermore, it is likely that the interventions will differ across health professionals and organizations, if these have different determinants of practice for implementation.

### Develop research protocols

We will plan rigorous evaluations of tailored implementation interventions in each of the different chronic diseases. The resulting tailored interventions may differ across the targeted chronic conditions. International standardization will be sought in order to enhance the comparability of research. The size of the studies will be determined on the basis of statistical power calculations, but we expect to include large samples of patients (>100 and potentially many more) and providers (>30 and potentially many more). Baseline measurements and patient outcome measures will be included when possible. Control groups will receive minor or delayed implementation interventions (in parallel group designs), or alternative interventions (in block designs). The trials will be pragmatic, meaning that they reflect clinical reality reasonably well.

In the analysis, we will examine the influence of the hypothesized determinants of practice on implementation processes in chronic illness care, and thus provide further evidence on the validity of measures of determinants of practice. This will provide hypotheses on factors that really influence implementation processes in chronic illness care. We will also test the added value of repeated analyses of determinants of practice compared to the initial analysis of determinants of practice. The data-analysis will be based on up-to-date tools, including random coefficient regression models for longitudinal data.

### Process evaluations

Process evaluations are especially necessary in trials of complex interventions, and in multisite trials, where the same or similar interventions may be implemented. In the process evaluation, we will measure the integrity and feasibility of the implementation interventions and clinical interventions that were implemented. Furthermore, we will assess how the interventions are implemented, distinguish between components of the interventions, and identify contextual factors that may influence the content and effectiveness of the implementation intervention. For this purpose, we will keep a log and use questionnaires for participants. The process evaluation will also examine how well the interventions were tailored to the local barriers/enablers, and whether the barriers were overcome to any extent. This will be based on interviews and surveys of participants. Process evaluation will consist of qualitative and quantitative methods that will be developed with this work package. The research teams in each country will apply those methods (*e.g*., central questions for interviews and focus groups) for process evaluation.

### Outcome evaluations

An outcome evaluation will assess the effectiveness of the tailored implementation interventions and the role of the previously identified determinants of practice in the implementation process. This analysis will also provide insight into the mechanisms underlying implementation processes in chronic illness care. The evaluations will include measures regarding the following domains: (intermediate) health outcomes including clinical measures, quality of life measures, and patient reported outcomes depending on the specific disease; patient behaviours, including adherence to treatment and life style); as well as healthcare professionals' performance and processes of healthcare delivery. Furthermore, descriptive information on patients, health professionals, and practice organizations will be systematically collected using questionnaires or medical records.

## Discussion

Across the world, health research funders have made substantial investments in implementation programs and in implementation research [7]. These activities should be led by the priorities in clinical practice and health policy, but it also important to invest in the assessment and innovation of approaches for implementation. The TICD project aims to contribute to the innovation of tailoring methods. The TICD project is focused on chronic illness care, which is a large and growing domain of healthcare across the world. It remains to be seen to what extent tailoring approaches are specific for this domain or can be used in other healthcare domains.

## Competing interests

Martin Eccles is Co-Editor in Chief of Implementation Science, Michel Wensing is an Associate Editor, Andy Oxman and Jeremy Grimshaw are members of the Editorial Board of Implementation Science; all decisions on this paper were made by another editor.

## Author's contributions

The study was conceived by MW. Writing of the paper was led by MW with all authors commenting on drafts and approving the final version.
